# Clinical role of echocardiography in LVAD outflow graft abnormalities: redefining the diagnostic paradigm

**DOI:** 10.1093/ehjimp/qyag032

**Published:** 2026-03-09

**Authors:** Vincenzo Nuzzi, Manlio Cipriani, Erberto Carluccio, Federico Fortuni

**Affiliations:** Clinical Cardiology and Heart Failure Unit, Mediterranean Institute for Transplantation and Advanced Specialized Therapies, ISMETT—IRCCS, Via Tricomi 5, Palermo 90127, Italy; Cardiothoracic Surgery Department, Heart and Vascular Centre, Maastricht University Medical Centre, Cardiovascular Research Institute Maastricht (CARIM), 6229 ER Maastricht, The Netherlands; UPMC Italy, Palermo, Italy; Clinical Cardiology and Heart Failure Unit, Mediterranean Institute for Transplantation and Advanced Specialized Therapies, ISMETT—IRCCS, Via Tricomi 5, Palermo 90127, Italy; UPMC Italy, Palermo, Italy; Cardiology and Cardiovascular Pathophysiology, Santa Maria Della Misericordia Hospital and University of Perugia, Perugia 06129, Italy; Cardiology and Cardiovascular Pathophysiology, Santa Maria Della Misericordia Hospital and University of Perugia, Perugia 06129, Italy

**This editorial refers to ‘Diagnostic accuracy of transthoracic echocardiography to detect structural abnormalities of the outflow graft in patients with left ventricular assist devices’, by T. Sato *et al*., https://doi.org/10.1093/ehjimp/qyag017.**

Advanced heart failure (AdvHF) remains a major clinical challenge. Although the adoption of optimal medial therapy, cardiac resynchronization therapy and interventional treatment of secondary mitral regurgitation (e.g. transcatheter edge-to-edge repair) has improved outcomes in chronic heart failure (HF), their benefit appears limited in AdvHF, in which overall prognosis remains poor.^1,2^ In this setting, long-term mechanical circulatory support with left ventricular assist devices (LVAD) has become a cornerstone of therapy, particularly given the shortage of donor organs and the presence of contraindications to heart transplantation in specific patients, such as severely non-reversible elevated pulmonary vascular resistance or active malignancy.^3^ LVAD therapy is associated with improvements in both survival and quality of life, underscoring the importance of accurate long-term care.^4^ While several common complications—such as gastrointestinal bleeding, superficial driveline infections, or cerebrovascular events—often present with a clear clinical onset and have a relatively straightforward diagnostic protocol, outflow graft (OG) abnormalities represents a more insidious complication. This issue may remain clinically silent at the beginning, as the flow is still sufficient to unload the left ventricle and maintain an adequate peripheral perfusion. Moreover, in the first phase, changes in LVAD parameters are often subtle and non-specific, especially if log files are not meticulously assessed, and major changes become evident only once the abnormality/obstruction has progressed to an advanced stage. Currently, the diagnosis of OG obstruction largely relies on structural imaging [i.e. contrast-enhanced computed tomography (CT)], and, in selected cases, invasive angiography and pressure gradient measurement^5,6^ However, this approach is not suitable for routine surveillance because of cumulative radiation exposure, repeated administration of iodinated contrast agents, invasive tests, and limited availability. In this view, transthoracic echocardiography (TTE) could support the diagnostic process and longitudinal follow-up of patients with LVAD, but its role remains incompletely defined, requiring further standardization and validation.

In this issue of European Heart Journal—Imaging Methods and Practice, Sato *et al*. report the interesting results of a retrospective analysis of 62 patients with LVAD, evaluating the diagnostic accuracy of TTE in detecting OG abnormalities, using CT as the reference gold standard (reference paper Sato *et al*. in press.^^7^^). First, feasibility of OG visualization was very high, exceeding that reported in prior cohorts, where feasibility ranged between 71% and 90%.^8,9^ In addition, specificity was excellent, reaching 100%, whereas sensitivity was only moderate (61%). However, sensitivity improved when the analysis was restricted to the most common site of obstruction with current devices, namely the proximal segment of the OG, increasing from 61% overall to 71%. When diagnostic performance was examined separately according to the underlying mechanism—stenosis, which predominates with contemporary devices, vs. graft bending—no significant differences were observed. The relatively limited sensitivity of TTE may reflect both the preserved flow velocities in the very early stages of obstruction, and technical challenges related to Doppler alignment. Notably, the prevalence of OG obstruction in this cohort was higher than that reported in previous larger series, a finding that is likely explained by a selection bias, as patients with clinical suspicion of obstruction are more likely to undergo chest CT and TTE. Importantly, this selection bias should not directly affect estimates of sensitivity and specificity. In contrast, the inclusion of different devices -HeartMate II, HeartMate 3, Jarvik 2000, HVAD, and EVAHEART 2—and, consequently, different OG configurations with inherently different physiological flow velocities may have diluted the results. Indeed, a uniform peak velocity cut-off of 2.0 m/s was applied, independently from the specific device tested. This threshold is slightly lower than normal values suggested for HVAD (2.07 ± 0.762 m/s) and higher than those for HeartMate II (1.74 ± 0.575 m/s) and HeartMate 3 (1.54 ± 0.46 m/s) devices, highlighting the challenge of defining universal cut-offs.^6^ A previous report in LVAD patients with symptomatic HF due to OG obstruction, using invasive gradient assessment, described a mean peak OG gradient of 78 mmHg, corresponding to an estimated velocity of ∼4.4 m/s. This finding suggests that lower Doppler velocity thresholds may enable earlier identification of OG abnormalities, before significant impairment of LVAD flow and systemic/pulmonary haemodynamics becomes clinically evident.^9^

In the evaluation of OG abnormalities, contrast-enhanced CT and invasive techniques remain the reference standard. TTE should not be viewed as a substitute for CT, but rather as a tool to redefine the diagnostic paradigm (*Figure 1*). Indeed, TTE is an early, non-invasive, low-cost, and widely available tool to support clinical decision-making and better justify the indication for second-level imaging or invasive tests. TTE is particularly useful for follow-up. Although reference ranges for pulsed-wave Doppler velocities in the OG have been proposed for different devices, substantial inter-individual variability exists in routine clinical practice.^6^ OG velocities are influenced by multiple factors, including residual left ventricular contractility, aortic valve opening, systemic blood pressure, and intravascular volume status.^10^ Consequently, absolute cut-off values may be less informative than patient-specific trends over time. A meaningful deviation from an individual patient’s baseline Doppler profile, especially when accompanied by clinical findings or device-related parameters suggestive of obstruction, should suggest closer surveillance or further diagnostic evaluation. Prospective studies focusing on contemporary cohorts of patients with LVAD are needed to define robust device-specific velocity threshold and to clarify their relationship with the severity and progression of any OG abnormalities. Most available studies have focused on peak systolic OG Doppler velocity. However, this parameter can be markedly influenced by left ventricular hemodynamics, including residual contractility, aortic valve opening, the presence of mitral regurgitation and diastolic dysfunction. From a pathophysiological perspective, assessment of diastolic OG flow may be less dependent on these factors and could therefore provide complementary and potentially more stable information. Large, multicentre cohorts will be essential to overcome the limitations inherent to single-centre experiences, including hospital protocol heterogeneity and technical challenges related to Doppler alignment, and to establish reliable and generalizable cut-offs. From this perspective, the implementation of AI-guided transthoracic echocardiography (TTE) imaging protocols^11^ to standardize the assessment of OG flow could help overcome key limitations related to Doppler alignment and operator dependency during image acquisition. Finally, achieving sufficient diagnostic sensitivity would allow TTE to be integrated as a reliable screening modality for OG obstruction, alongside clinical assessment and device-derived parameters.

**Figure 1:**
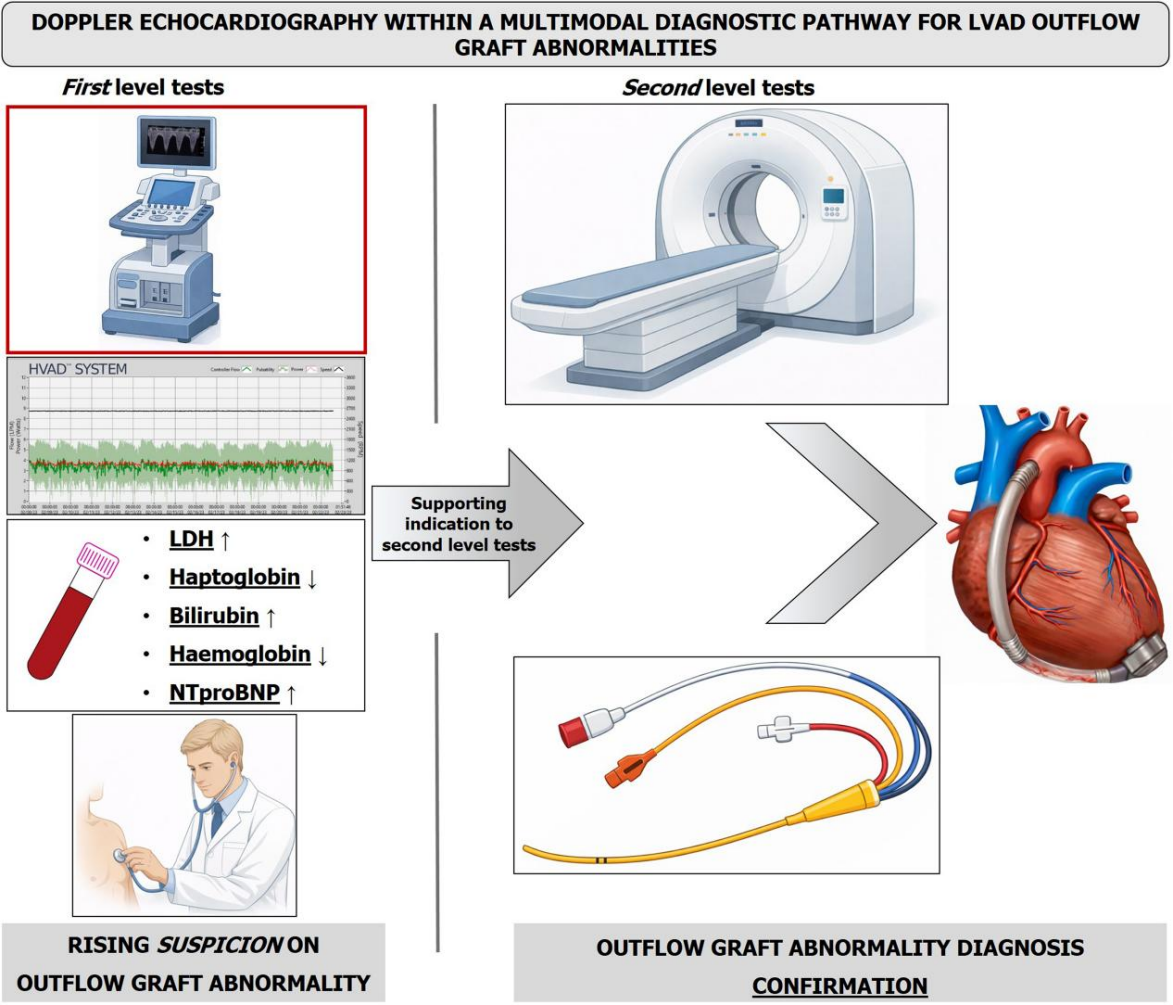
Non-invasive findings, including transthoracic echocardiography with Doppler assessment of the outflow graft, LVAD log file analysis, clinical findings, and laboratory parameters, should raise suspicion of outflow graft abnormalities, supporting the indication to second-level diagnostic tests. Contrast-enhanced computed tomography and invasive haemodynamic assessment provide anatomical and functional confirmation of the abnormality, allowing definitive diagnosis and guiding management.

## Data Availability

No new data were generated or analysed in support of this research.
